# A comparative analysis of surface and bulk contributions to second-harmonic generation in centrosymmetric nanoparticles

**DOI:** 10.1038/s41598-018-21850-8

**Published:** 2018-02-26

**Authors:** Daniel Timbrell, Jian Wei You, Yuri S. Kivshar, Nicolae C. Panoiu

**Affiliations:** 1 Department of Electronic and Electrical Engineering, University College London, Torrington Place, London, WC1E 7JE United Kingdom; 2 Nonlinear Physics Centre, Australian National University, Canberra, ACT 2601 Australia

## Abstract

Second-harmonic generation (SHG) from nanoparticles made of centrosymmetric materials provides an effective tool to characterize many important properties of photonic structures at the subwavelength scale. Here we study the relative contribution of surface and bulk effects to SHG for plasmonic and dielectric nanostructures made of centrosymmetric materials in both dispersive and non-dispersive regimes. Our calculations of the far-fields generated by the nonlinear surface and bulk currents reveal that the size of the nanoparticle strongly influences the amount and relative contributions of the surface and bulk SHG effects. Importantly, our study reveals that, whereas for plasmonic nanoparticles the surface contribution is always dominant, the bulk and surface SHG effects can become comparable for dielectric nanoparticles, and thus they both should be taken into account when analyzing nonlinear optical properties of all-dielectric nanostructures.

## Introduction

The strong field enhancement that accompanies the excitation of surface-plasmon polaritons (SPPs) on metallic nanoparticles^1,2^ makes these nanostructures ideal candidates for many applications, including nanoscale antennae, single-molecule detection *via* surface-enhanced Raman scattering, metallic nanotips for near-field optical microscopy, and optically-active guiding nanostructures^3–9^. However, the generation of large optical fields comes at a price of significant optical losses present in all metals. In fact, these losses are viewed as the main factor that still precludes a widespread use of plasmonic devices in practical applications^10^. Methods that attempt to overcome this restriction include utilizing doped semiconductors^11^ and optical gain media^12^.

An alternative to plasmonic materials, which aims to circumvent optical losses, consists of using all-dielectric structures^13,14^. Unlike the excitation of the metallic plasma that engenders the plasmonic resonances, it is the resonances of displacement currents, known as *Mie resonances*^15^, that enable these all-dielectric components to be used for optical field manipulation. While the field enhancement of these dielectric structures is typically weaker than that of their metallic counterparts, their high quality factors enable intriguing optical phenomena to be produced, including magnetic mirrors^16^, reflectionless ultrathin sheets mimicking highly directional Huygens sources^17,18^, and toroidal dipole sources^19,20^. These non-metallic nanostructures have shown great promise in biosensing, optoelectronics, and energy applications^21^. Hence, the trade-off is clear: the selection of metal or dielectric is dependent upon the requirements of particular applications, namely whether one desires strong field enhancement or low optical losses.

This dichotomy extends to *nonlinear nanoscale photonics*. By exciting a structure that has both strong field enhancement potential and strong nonlinear properties, large nonlinear signals can be produced at relatively low optical powers. In particular, nonlinear optical processes in plasmonic structures have been studied extensively^22,23^, including surface-enhanced Raman scattering^3,7,24^, second-order optical interactions^25–36^, and Kerr interactions^37,38^. As in the linear case, these strong nonlinear optical effects in plasmonic structures are accompanied by large optical losses, which restrict the range of applications to which nonlinear optical interactions can be employed. It is therefore of particular interest to understand, in the context of nonlinear nanophotonics, the limitations and advantages provided by plasmonic structures, as compared to those characteristic to all-dielectric resonant subwavelength structures.

In order to address this important problem, in this paper we focus on second-harmonic generation (SHG), perhaps the most widely studied nonlinear optical interaction. Since most plasmonic materials are centrosymmetric, i.e. the crystal lattice is invariant upon inversion symmetry transformations, we consider for comparison dielectric materials that are also centrosymmetric. More specifically, we assume that the plasmonic and dielectric materials are *gold* (Au) and *silicon* (Si), respectively. Under these circumstances, the second-harmonic (SH) field has two principal components, namely the (local) surface and (nonlocal) bulk contributions of the medium to a nonlinear signal. From a physical point of view, as will become clear from the mathematical description of these nonlinear optical effects, the main difference between the two contributions is that whereas the surface component is (quadratically) proportional to the optical field at the fundamental frequency (FF), the bulk component is proportional to the field and its derivatives.

It is a common assumption that the bulk nonlinear response to an applied electric field is negligible for plasmonic structures. The validity of this assumption, however, has hitherto been rigorously addressed only for plasmonic structures with simple configurations, such as thin-films^39^, spherical particles^40^, and split-ring resonators^41^, *no attempts having been made to investigate this problem in the case of dielectric particles*. It should be noted that this problem does not have a simple, *a priori* answer. Thus, in plasmonic materials optical fields are highly inhomogeneous, so that the field derivatives can be very large; however, these fields extend from the surface into the bulk no more than about a skin-depth, i.e., the characteristic distance the electric field penetrates into a metal. By contrast, optical fields penetrate throughout a dielectric structure, yet they are much less inhomogeneous as compared to the plasmonic case. To elucidate this matter, in this paper we study theoretically and computationally, the relative contribution to SHG of the surface and bulk effects in two generic structures made of centrosymmetric materials, one metallic and one dielectric, both exhibiting resonant field enhancement. In particular, we choose cruciform-shaped particles as they have pronounced electric dipole, magnetic dipole, and electric quadrupole resonances. However, more complex resonances, such as Fano resonances^42–45^, or particle shapes can be considered.

## Model and scattering configuration

**Figure 1:**
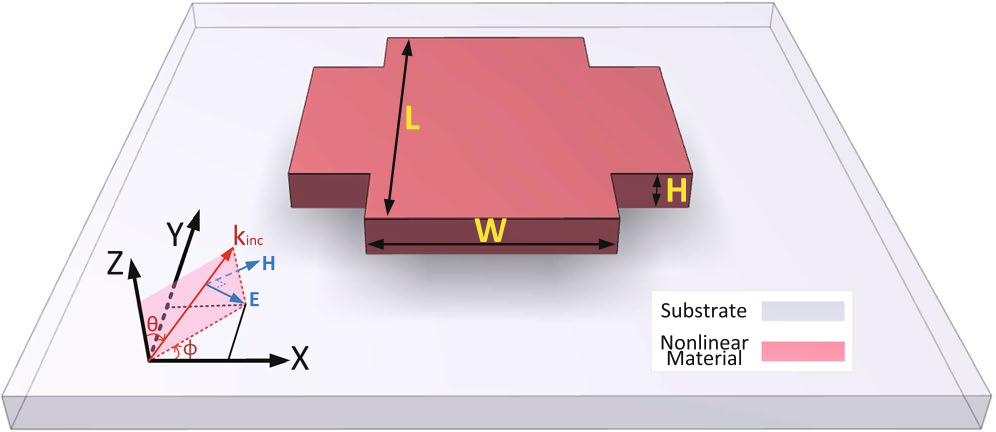
Schematics of the analyzed structure. A cruciform scatterer made of centrosymmetric materials (Au or Si) sits atop a glass substrate lying in the *xy*-plane. The gold cross has dimensions of *L* = 100 nm, *W* = 55 nm, and *H* = 30 nm, whereas the silicon cross has dimensions of *L* = 370 nm, *W* = 220 nm, and *H* = 220 nm. The cross is illuminated with a plane wave impinging normally onto the substrate, with the electric and magnetic fields being oriented along the arms of the cross. Hence, for the purposes of our calculations, we set *θ* = *π* and *ϕ* = *π*/2.

The structure analyzed here is shown schematically in Fig. 1, with the dimensions given in the caption. This symmetric cross sits atop a glass substrate, with permittivity *ε*_*s*_ = 2.25, which is assumed to occupy the region *z* < 0. The cross is illuminated with normal-incidence light (-*z*-direction), with the **E** and **H** fields polarized along the *x*- and -*y*-direction, respectively, the corresponding wave intensity being 1 GW/cm^2^. The optical response of this structure is, however, polarization-independent due to its symmetry.

The choice of dimensions of the particles was guided by general characteristics of common experimental set-ups. Thus, plasmonics experiments are usually performed in the visible spectrum, whereas most applications of silicon devices are in the IR spectral domain (1.3 µm and 1.55 µm for data centers and telecom applications, respectively). Therefore, we chose the dimensions of our particles such that they have resonances in the corresponding spectral domain of practical interest. In addition, we chose a thickness of the silicon cross of *H* = 220 nm because a common material platform employed in nonlinear optics experiments is silicon-on-insulator (SOI), the thickness of the silicon layer of commonly used SOI wafers being *H* = 220 nm.

We select the cruciform shape of particles as they are an excellent middle-ground between analytically solvable structures such as spheres and specifically tailored structures, i.e. they are complex enough to provide generality to our conclusions yet not too complex to completely obscure the origin of the revealed physics. In addition, the cruciform particles support the most basic Mie-type resonances, i.e. electric dipole, magnetic dipole, and electric quadrupole resonances. Therefore, we expect that most of the new physics revealed by our study should apply to nanoparticles with other shapes, too, as there is nothing specific regarding the nature of the Mie resonances of the nanoparticles investigated in this work. Finally, one expects that the ratio between the contribution of bulk and surface effects to SHG is primarily determined by the inhomogeneity of the optical near-field at the FF, an effect that is more predominant at the nanoparticle resonances. This inhomogeneity is the largest at the wavelengths of the resonances of the particle, and does not change significantly with the angle of incidence. Therefore, we employ a simple scattering configuration, which still captures the main physics of the problem investigated in our study.

The linear optical response of the cruciform structures has been calculated using the time-domain solver of CST Studio Suite^46^. Periodic boundary conditions are employed in the *x*- and *y*-direction, whereas in the *z*-direction we use perfectly match layers so that no waves are reflected back into the computational domain. The period is chosen large enough so as in the spectral range considered here the optical coupling between the crosses is negligible and thus we can assume that we operate in the single-scatterer regime. Moreover, in order to study size dependence effects, we also consider crosses whose arm length and width are scaled by a factor *α* (the height is kept constant, as this choice better reflects standard experimental conditions), with *α* = 1, 1.25, 1.5, 1.75 and 2.

In our calculations, a frequency scan from 200-1000 THz (150-500 THz) in 4 THz (0.5 THz) increments is performed for gold (silicon), for a sufficiently fine computational mesh. In both cases the frequency dispersion of the permittivity is fully taken into account by fitting the experimental data with a Drude function and a set of Lorentzians in the case of Au, and a set of Lorentzians in the case of Si (see Supporting Information for more details regarding the modeling of the frequency dispersion of Au and Si). Note that our models for the frequency dispersion of the permittivity of gold and silicon take into account both interband transitions effects in the case of gold and bandgap effects in the case of silicon.

As is well known, electromagnetic multipoles associated to a nanoparticle can reveal key physical insights into the optical properties of the scatterer^47^. Therefore, for both types of crosses, we performed a multipole decomposition, whereby the radiated powers associated with the first three terms of the multipole expansion were calculated. These multipoles, the electric dipole, magnetic dipole, and electric quadrupole, are defined as

1a $${{\bf{p}}}_{\omega }=\int {{\bf{P}}}_{\omega }({\bf{r}})d{\bf{r}},$$

1b $${{\bf{m}}}_{\omega }=-i\frac{\omega }{2}\int {\bf{r}}\times {{\bf{P}}}_{\omega }({\bf{r}})d{\bf{r}},$$

1c $${Q}_{\omega ,jk}=\int \mathrm{[3(}{r}_{j}{P}_{\omega ,k}+{r}_{k}{P}_{\omega ,j})-2{\delta }_{jk}{\bf{r}}\cdot {{\bf{P}}}_{\omega }({\bf{r}})]d{\bf{r}},$$

where *P*_*ω*_ = *ε*_0_[*ε*_*r*_(*ω*) − 1]*E*_*ω*_(*r*) is the polarization of the medium at the FF, *ω*, at position *r*, dependent upon the electric field *E*_*ω*_(*r*), with *ε*_0_ and *ε*_*r*_ denoting the vacuum permittivity and the relative permittivity of the medium, respectively. It is important to note that Eqs. (1) incorporate the conductive and displacement currents if the dispersive character of *ε*_*r*_ is taken into account. The power radiated by this collection of multipoles is calculated *via* their far-field Poynting flux. We restrict ourselves here to analyzing the currents engendered within the cross only.

In order to describe the nonlinear optical signal generated by the scatterer, we employ a widely used model of SHG in centrosymmetric media^48^. Specifically, the SHG has a surface component generated within a few atomic layers at the surface of the material and a bulk component generated inside the material. The surface nonlinear polarization is described by a surface nonlinear susceptibility and can be written as:

2 $${{\bf{P}}}_{{\rm{\Omega }}}^{s}({\bf{r}})={\varepsilon }_{0}{\hat{\chi }}_{s}^{\mathrm{(2)}\,}:\,{{\bf{E}}}_{\omega }({\bf{r}}){{\bf{E}}}_{\omega }({\bf{r}})\delta ({\bf{r}}-{{\bf{r}}}_{s}),$$

where Ω = 2*ω* is the SH frequency, ***r***_*s*_ defines the surface, ${\hat{\chi }}_{s}^{\mathrm{(2)}}$ is the surface second-order susceptibility tensor, and the Dirac delta-function expresses the surface characteristic of the nonlinear polarization. Note that in Eq. (2) the fields are evaluated just inside the nanoparticle^39,48,49^.

Except for the case when the surface contains structural features with intrinsic chirality, the surface of centrosymmetric media possesses an isotropic mirror-symmetry plane perpendicular to the interface. Then, the surface nonlinear susceptibility ${\hat{\chi }}_{s}^{\mathrm{(2)}}$ has only three independent components, that is, ${\hat{\chi }}_{s,\perp \perp \perp }^{\mathrm{(2)}}$, ${\hat{\chi }}_{s,\perp \parallel \,\parallel }^{\mathrm{(2)}}$, and ${\hat{\chi }}_{s,\parallel \perp \parallel }^{\mathrm{(2)}}={\hat{\chi }}_{s,\parallel \parallel \perp }^{\mathrm{(2)}}$, where the symbols ⊥ and $\parallel$ refer to the directions normal and tangent to the surface, respectively. As most theoretical models predict that ${\hat{\chi }}_{s,\perp \parallel \parallel }^{\mathrm{(2)}}=0$^50,51^, we make this assumption in our calculations, too. The susceptibility components of Au, measured at *λ* = 810 nm, are ${\hat{\chi }}_{s,\perp \perp \perp }^{\mathrm{(2)}}=-(0.86+1.34i)\times {10}^{-18}\,{{\rm{m}}}^{2}{{\rm{V}}}^{-1}$ and ${\hat{\chi }}_{s,\parallel \parallel \perp }^{\mathrm{(2)}}={\hat{\chi }}_{s,\parallel \perp \parallel }^{\mathrm{(2)}}=-(4.61+0.43i)\times {10}^{-20}\,{{\rm{m}}}^{2}{{\rm{V}}}^{-1}$ ^52^, whereas in the case of Si, their measured values at *λ* = 800 nm are ${\hat{\chi }}_{s,\perp \perp \perp }^{\mathrm{(2)}}=65\times {10}^{-19}{{\rm{m}}}^{2}{{\rm{V}}}^{-1}$ and ${\hat{\chi }}_{s,\parallel \parallel \perp }^{\mathrm{(2)}}={\hat{\chi }}_{s,\parallel \perp \parallel }^{\mathrm{(2)}}=3.5\times {10}^{-19}{{\rm{m}}}^{2}{{\rm{V}}}^{-1}$ ^53^.

The second component of the nonlinear polarization is generated in the bulk of the material and is written as:

3 $${{\bf{P}}}_{i,{\rm{\Omega }}}^{b}({\bf{r}})=\gamma {\nabla }_{i}[{{\bf{E}}}_{\omega }({\bf{r}})\cdot {{\bf{E}}}_{\omega }({\bf{r}})]+{\delta }^{\text{'}}[{{\bf{E}}}_{\omega }({\bf{r}})\cdot \nabla ]{{\bf{E}}}_{i,\omega }({\bf{r}})+\beta {{\bf{E}}}_{i,\omega }({\bf{r}})[\nabla \cdot {{\bf{E}}}_{\omega }({\bf{r}})]+\zeta {{\bf{E}}}_{i,\omega }({\bf{r}}){\nabla }_{i}{{\bf{E}}}_{i,\omega }({\bf{r}}),$$

where *γ*, *δ*^′^, *β*, and *ζ* are material parameters. This (nonlocal) polarization originates from electric quadrupoles and magnetic dipoles located in the bulk. This is the dominant bulk contribution in centrosymmetric media, as in this case the contribution of electric dipoles vanishes.

The third term in Eq. (3) can usually be neglected, as in homogeneous media ∇ ⋅ ***E***_*ω*_(***r***) = 0. Also, most theoretical models^54^ predict that the second term is negligible, too. For example, in the case of plane wave propagation in a homogeneous medium this term exactly cancels due to the transverse character of the field at the FF. Moreover, in the case of noble metals the ratio between *δ*^′^ and *γ* is of the order of *ν*/*ω*^39^, where *ν* is the damping frequency, and this ratio is negligible at optical frequencies. We note, however, that the degree to which the term proportional to *δ*^′^ influences the SHG is still a matter of debate^39^. Based on these considerations, in this work we set *δ*^′^ = 0 and neglect the third term in Eq. (3). Furthermore, in the case of noble metals, the anisotropy parameter has a negligible value, so that in our calculations we set *ζ* = 0 for Au, whereas for Si, at *λ* = 800 nm, $\zeta =2.3\times 1{0}^{-19}{{\rm{m}}}^{2}{{\rm{V}}}^{-1}$ ^53^. The values of *γ* for the two materials considered in this study are $\gamma =7.13\times {10}^{-21}{{\rm{m}}}^{2}{{\rm{V}}}^{-1}$ (Au at *λ* = 810 nm)^52^ and $\gamma =1.3\times {10}^{-19}{{\rm{m}}}^{2}{{\rm{V}}}^{-1}$ (Si at *λ* = 800 nm)^53^.

Since experimental data for the frequency dispersion of the surface and bulk nonlinear susceptibilities is not available, we used the Miller rule^55^ to incorporate this property of the nonlinear susceptibilities in our analysis. It should describe rather well the frequency dispersion of the nonlinearity of Si in the IR and mid-IR because in this frequency domain the linear frequency dispersion of Si is rather small (see also Supporting Information) yet the use of this rule in the case of Au might be somewhat problematic due the influence of interband transitions. It should be stressed that we do not apply the Miller rule to the nonlinear response of the nanoparticle, as in such a case its predictions are unreliable^56^, but to the *intrinsic material optical constants* of Au and Si. According to the Miller rule, the following relation holds,

4 $$\frac{{\chi }^{\mathrm{(2)}}({\rm{\Omega }},\omega )}{{\chi }^{\mathrm{(1)}}({\rm{\Omega }}){[{\chi }^{\mathrm{(1)}}(\omega )]}^{2}}={\mathscr{C}},$$

where *χ*^(2)^(Ω, *ω*) and *χ*^(1)^(*ω*) are the nonlinear and linear susceptibilities, respectively, and ${\mathscr{C}}$ is a constant. Thus, knowing *χ*^(2)^(Ω, *ω*) and *χ*^(1)^(*ω*) at a particular wavelength allows us to calculate the constant ${\mathscr{C}}$ and subsequently the nonlinear susceptibility *χ*^(2)^(Ω, *ω*) at any frequency. More details about these calculations can be found in the Supporting Information.

The nonlinear polarizations in Eqs (2) and (3) can be used to calculate the nonlinear multipoles *via* Eq. (1), which can be viewed as multipolar sources for the nonlinear field. However, this approach is less accurate when applied to the SH calculations, as in this case the ratio between the nanoparticle size and wavelength is larger than it is at the FF and therefore the multipole expansion converges more slowly. This is illustrated by Fig. 7 in the Supporting Information. As a result, we use an alternative method to calculate the nonlinear optical response of the crosses. Thus, these same polarizations define nonlinear currents, *via* ${{\bf{J}}}_{\Omega }^{s,b}({\bf{r}})=-i{\rm{\Omega }}{{\bf{P}}}_{{\rm{\Omega }}}^{s,b}({\bf{r}})$ (an *e*^−*iωt*^ dependence of all harmonic fields is assumed throughout this study). These nonlinear currents are subsequently used to calculate the nonlinear optical far-field by employing a near-field/far-field transformation^57^, thus enabling a complete characterization of the nonlinear scattering process.

## Results and Discussion

**Figure 2:**
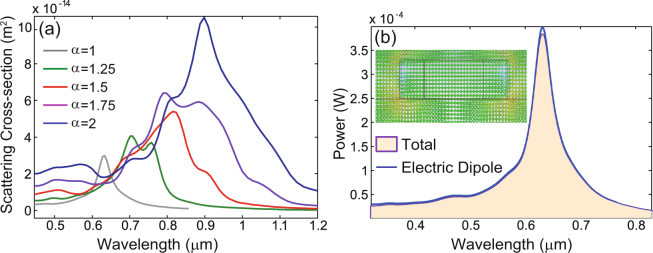
Linear spectral properties of gold crosses. (**a**) The spectra of the linear scattering cross-section of a cross made of gold, determined for different scaling values, *α*. (**b**) A more detailed understanding of the linear regime for the case *α* = 1 is provided by the spectra of the total radiated power and the power radiated by the electric dipole of the cross. The inset shows the electric field distribution calculated at the resonance wavelength in the *xz*-plane passing through the center of the cross.

The nonlinear polarization, and implicitly the sources of the SH field, is primarily determined by the field at the FF, and therefore we have started our analysis with the calculation of the linear optical field. The spectra of the linear scattering cross-section of the metallic crosses, determined for several values of the scaling parameter *α*, are plotted in Fig. 2(a). They show a prominent resonance, which for the cross with *α* = 1 is located at 0.63 µm. Unsurprisingly, as the cross is scaled to larger sizes, the resonance peak shifts to increasing wavelengths. The field distribution calculated at the resonance wavelength of the cross with *α* = 1 and shown in the inset of Fig. 2(b), suggests that this is an electric dipole resonance. To confirm this, we performed a multipole decomposition, as per Eq. (1). The results of these calculations, summarized in Fig. 2(b) for *α* = 1, clearly prove that the electric dipole has the dominant contribution to the total radiated power, in the entire wavelength range. The peak of this electric dipole spectrum is located at 0.63 µm, thus further validating our conclusion.

**Figure 3:**
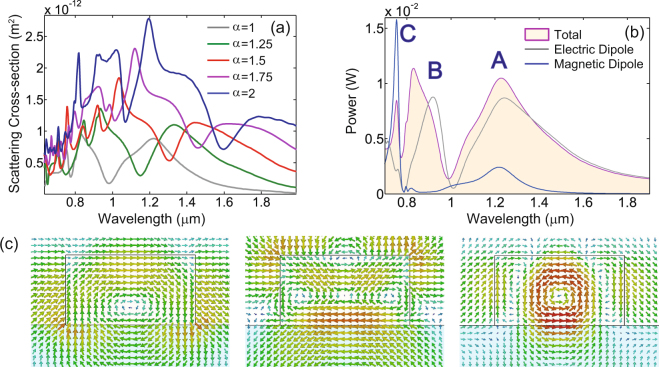
Linear spectral properties of silicon crosses. (**a**) The spectra of the linear scattering cross-section of a silicon cross, determined for different scaling values, *α*. (**b**) A more detailed understanding of the linear regime for the case *α* = 1 is provided by the spectra of the total radiated power and the power radiated by the electric dipole and magnetic dipole of the cross. (**c**) Near-field distributions determined at the main resonances. Resonances *A* and *C* are primarily of electric dipole and magnetic dipole type, respectively, whereas additional terms must be considered to accurately describe resonance *B*. Note also that there is a small contribution to resonance *A* from a magnetic dipole. From left to right, bottom panels show the electric field distribution at the resonances *A*, *B*, and *C*, in the *xz*-plane passing through the center of the cross.

We compare these fundamental field results to those corresponding to a cross made of Si. The spectra of the linear scattering cross-section of this structure, determined for the same five values of *α*, are shown in Fig. 3(a). Due to the dielectric nature of the cross, more spectral resonances exist within the scanned wavelength range. In particular, as the wavelength of the incoming light decreases, higher-order (Mie) resonances of the cross can be excited within the structure. Figure 3(b) shows the spectra of the total radiated power as well as the spectra corresponding to the electric dipole and magnetic dipole, all calculated for the cross with *α* = 1, whereas the resulting electric field distributions within the Si structure, calculated at the wavelengths of the first three resonances, namely at 0.73 µm, 0.81 µm, and 1.2 µm, are presented in Fig. 3(c) (additional information about the near-field distributions corresponding to these resonances is provided in Supporting Information Fig. 9). As these figures illustrate, the multipole expansion is less accurate when applied to the Si cross, chiefly because in this case the ratio between the cross size and wavelength is larger than that for the Au cross and thus the long-wavelength approximation, in which the multipole expansion holds, becomes inaccurate.

Inspecting the spectrum of the total radiated power, one can observe three distinct peaks within the wavelength range. The spectra in Fig. 3(b) and the field profiles shown in Fig. 3(c) suggest that peaks *A* and *C* correspond to an electric dipole mode and a superposition of a strong magnetic dipole mode and a weaker, higher-order magnetic mode, respectively, whereas the spectral peak *B* is the result of a mixture of multipole resonances. Note also that both the scattering spectra and field distributions suggest that at peak *A* there is an additional contribution from a magnetic dipole mode. As illustrated in the Supporting Information, additional information about the nature of these resonances can be obtained from the differential scattering cross sections calculated at the resonance wavelengths, as well as those associated to the electric dipole, magnetic dipole, and electric quadrupole, calculated at the same resonance wavelengths. Moreover, one can clearly see in Fig. 3(b) spectral regions where the light radiated by different multipoles interfere destructively or constructively, thus suppressing or enhancing the total radiated power, respectively. This interference among optical fields emitted by different multipoles of a scatterer has been observed experimentally^58^, both in the linear and nonlinear regimes.

It must be noted that, as expected, the quality factor of the resonances of the gold cross is smaller than that of the silicon cross. This is due to the optical losses in the metal, which add to the radiative ones. Moreover, the total power radiated by the silicon cross naturally outweighs the total power scattered by the gold cross, as the ratio of the structural volumes is *V*_*Si*_/*V*_*Au*_ = 108.5.

We move now on to the nonlinear optical response of the two types of crosses and start with a brief discussion of the key ideas pertaining to the basic nonlinear physics. The external field of frequency, *ω*, impinges on the “meta-atom”, exciting an electromagnetic field at frequency, Ω = 2*ω*. Hence, we expect to see a strong SH signal when optical resonances are excited at the FF. Generally, this nonlinear optical process can occur both at the surface and in the bulk of the scatterer, with different physics being responsible for each component, as described by Eqs (2) and (3).

**Figure 4:**
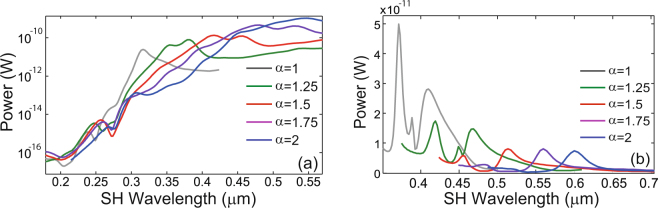
Spectral properties of surface second-harmonic generation in gold and silicon crosses. (**a**) The spectra of the power radiated at the second-harmonic by the nonlinear surface sources induced in a cross made of gold, determined for different scaling values, *α*. (**b**) The same as in (**a**) but calculated for crosses made of silicon. A log scale is chosen for the Au case to help highlight the resonances.

In order to calculate the surface and bulk nonlinear polarizations we used Eqs (2) and (3) in conjunction with the fields at the FF. These nonlinear polarizations are then employed to calculate the corresponding surface and bulk optical powers and the total power emitted at the SH, as explained in the preceding section. Since these calculations are performed in the frequency domain, this approach can be readily applied both in the case when the nonlinear coefficients are frequency dependent, as well as in the dispersionless case. The results of this analysis, determined for the surface component and for all of the Au and Si crosses, are plotted in Fig. 4. As is evident from this figure, the resonances observed at the SH are located at wavelengths Λ_*r*_ = *λ*_*r*_/2, where *λ*_*r*_ are the corresponding resonance wavelengths at the FF.

A more careful examination of the power spectra depicted in Fig. 4 reveals several differences between the nonlinear optical response of Au and Si crosses. Thus, with regards to the Au cross, the electric dipole generated at the FF is the main source of power radiated at the SH. Indeed, the wavelength of the maximum SH intensity coincides with half the wavelength of the dipole resonance at the FF (see Fig. 2). However, by contrast, in the case of the Si cross the electric dipole resonance has the weakest signature in the nonlinear spectrum, the main signal at the SH originating from the resonances labeled in Fig. 3(b) with *B* and *C* (magnetic dipole resonance). This suggests that in the case of the electric dipole resonance of the Si cross, there is only a small overlap between the distribution of nonlinear surface currents and the plane waves of the radiation continuum, and therefore only a small amount of light is radiated.

An equally important but rather unexpected result is that in the case of the silicon cross the strongest nonlinear signal is generated by the smallest cross. This is explained by the fact that as the size of the cross increases the resonance wavelength is red-shifted to a spectral region of much weaker surface nonlinearity (see Fig. 2 in the Supporting Information). This size dependence of the surface SHG becomes more complex in the case of the gold cross. Thus, Fig. 4(a) shows that the SHG increases as the size of the cross increases, whereas if the dispersion of the surface nonlinearity is neglected the opposite dependence is observed (see Supporting Information). There are two concurrent effects that lead to these outcomes. Thus, when the size of the cross increases the resonance wavelength increases, too, so that it moves in a spectral region of much larger surface nonlinearity. On the other hand, the size dependence of the surface SHG observed in the dispersionless case is explained by the fact that, as our numerical simulations and previous works corroborate^59^, the strongest field enhancement is achieved for the crosses with the smallest size, an effect that outweighs the fact that in this case the SH is generated on a smaller surface. More specifically, the maximum field enhancement computed for gold crosses with *α* = 1, 1.25, 1.5, 1.75, and 2 is 56.2, 44.6, 34.8, 27 and 20.5, respectively.

**Figure 5:**
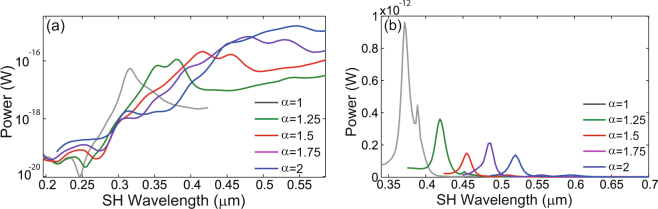
Spectral properties of bulk second-harmonic generation in gold and silicon crosses. (**a**) The spectra of the power radiated at the second-harmonic by the nonlinear bulk sources induced in a cross made of gold, determined for different scaling values, *α*. (**b**) The same as in (**a**) but calculated for crosses made of silicon. A log scale is chosen for the Au case to help highlight the resonances.

In order to quantify the relative contributions of the surface and bulk nonlinear currents to the total radiated power at the SH, we show in Fig. 5 the radiation spectra corresponding to the bulk nonlinear polarization, calculated for both types of crosses and for all values of the scaling factor. These calculations show that generally the bulk effects are much weaker than the surface ones, by more than four orders of magnitude for gold crosses and by more than two orders of magnitude for crosses made of silicon. In addition, it can be seen that the relative strength of the spectral resonances varies from the surface to bulk spectra, which is particularly evident in the case of Si crosses. In the Supporting Information we also plot in Fig. 10 the nonlinear surface and bulk currents calculated for a resonance wavelength at the FF, for the case *α* = 1.5 and for both types of crosses, to further illustrate these effects.

**Figure 6:**
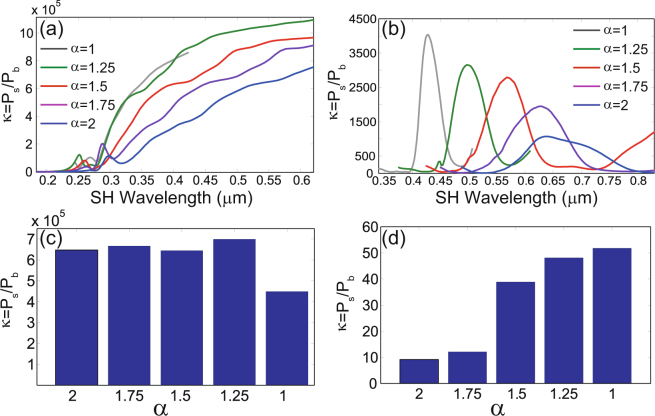
Relative contribution of surface and bulk to second-harmonic generation in gold and silicon crosses. (**a**) The dependence of the ratio *κ* = *P*_*s*_/*P*_*b*_ between the SH powers generated by the surface and bulk nonlinear sources induced in a cross made of gold, determined for different scaling values, *α*. (**b**) The same as in (**a**) but calculated for the crosses made of silicon. (**c**) The value of the ratio *κ* evaluated at the wavelength at which the bulk contribution is maximum vs. the scaling parameter *α*, calculated for the crosses made of gold. (**d**) The same as in (**c**) but calculated for the crosses made of silicon.

Despite the fact that generally the nonlinear bulk effects are weaker than the surface ones, close to bulk resonances their contribution can become comparable. Here we call bulk (surface) resonances spectral peaks that are observed when only bulk (surface) nonlinear currents are included in the calculations. In order to illustrate this important idea, we plot in Fig. 6(a) and (b) the ratio, *κ* = *P*_*s*_/*P*_*b*_, between the SH powers generated by the surface and bulk nonlinear currents, *P*_*s*_ and *P*_*b*_, respectively, determined for the gold and silicon crosses, and for all five scaling factors. These figures clearly show that in the long-wavelength limit the bulk contribution to the total SHG is negligible. For wavelengths comparable to the size of the crosses, however, the bulk contribution can become commensurate to that of the surface, but only for silicon crosses. Equally important, even though the surface and bulk contributions to SHG are different in the dispersive and dispersionless cases, their ratio is almost the same (compare Fig. 6 in the paper and Fig. 5 in Supporting Information). This conclusion is explained by the fact that, due to the Miller rule, both contributions scale with frequency in the same way.

The surface and volume of the crosses increase at different rate with the cross size and therefore one expects that the relative contribution of the surface and bulk nonlinear currents varies with the size of the crosses. In order to verify this argument, we evaluated the ratio *κ* at the wavelength at which the bulk contribution is the largest and repeated these calculations for all values of the scaling factor, both for the gold and silicon crosses. The histograms corresponding to the gold and silicon crosses, presented in Fig. 6(c) and (d), respectively, suggest that there are significant qualitative differences between the two cases. Thus, in the case of metallic crosses, *κ* does not vary much with *α*. This apparent paradox is explained by the fact that the effective region where nonlinear currents are induced in the bulk only extends about a skin-depth from the surface into the metal (see also Fig. 8 in Supporting Information). As a result, the surface and bulk effects increase in fact at the same rate with the size of the gold cross, and therefore the ratio *κ* should only weakly depend on *α*. In the case of dielectrics, on the other hand, the field at the FF penetrates throughout the (nonlinear) medium and therefore nonlinear currents are induced in the entire bulk region. Consequently, the bulk part increases faster with the cross size as compared to the surface one, in complete agreement with the dependence of *κ* on *α* shown in Fig. 6(d). All these conclusions remain valid in the non-dispersive case, too, which can be inferred from Fig. 5(c) and (d) in Supporting Information.

We stress that we did not attempt to optimize our structures so as to maximize the contribution of the bulk effects to the SHG. Nevertheless, our analysis suggests that it is conceivable that, at least in the case of all-dielectric nanoparticles, one can design structures for which the bulk effects are comparable or even larger than the surface ones. This means that care must be taken when experimental results pertaining to SHG in all-dielectric nanostructures made of centrosymmetric materials are theoretically interpreted, as our analysis suggests that the validity of the commonly used practice to neglect the bulk contribution to SHG might break down in this instance.

## Conclusion

We have studied the second-harmonic generation arising from cruciform structures made of centrosymmetric metallic and dielectric materials. We have focused on the nonlinear optical response of such subwavelength scatterers, aiming to elucidate whether the surface or bulk contribution to the second-harmonic generation is dominant. Our analysis has provided a nuanced answer to this question, namely in the case of metallic structures the nonlinear power generated by surface interactions surpasses *by orders of magnitude* that due to bulk effects, whereas in dielectric structures, in certain circumstances, these two contributions can become comparable. We have also discussed practical implications of these findings for subsequent experimental verifications.

## Electronic supplementary material

Supplementary Information
