# Supplementary material for: A comparative analysis of surface and bulk contributions to second-harmonic generation in centrosymmetric nanoparticles
Source: Sci Rep. 2018 Feb 26;8:3586. doi: 10.1038/s41598-018-21850-8 (PMC5826928; doi:10.1038/s41598-018-21850-8)
Supplement: Supplementary file 1 — Supplementary Information [file 41598_2018_21850_MOESM1_ESM.pdf]

SUPPORTING INFORMATION

A comparative analysis of surface and bulk contributions to second-harmonic generation in centrosymmetric nanoparticles

Daniel Timbrell[1], Jian Wei You[1], Yuri S. Kivshar[2], and Nicolae C. Panoiu[1,*]

[1]Department of Electronic and Electrical Engineering,University College London, Torrington Place, London WC1E 7JE, United Kingdom
[2]Nonlinear Physics Centre, Australian National University, Canberra, ACT 2601, Australia
[*]n.panoiu@ucl.ac.uk

ABSTRACT

The frequency dispersion of linear susceptibilities of Au and Si are presented, as well as the frequency dispersion of their surface and bulk second-order susceptibilities. We also provide the linear differential scattering cross-section of crosses made of Au and Si and the near-field distribution calculated at specific resonance wavelengths.

Frequency Dispersion of Linear Susceptibility of Au and Si

A key property of optical materials is the frequency dispersion of their permittivity. This wavelength-dependent function describes the electromagnetic response of the material in the linear regime. Figures 1(a) and 1(b) depict the real and imaginary parts of the susceptibility function, $\chi^{(1)}$, of Au and Si, respectively. As can be seen by comparing the data plotted in Fig. 1, the electric response of the metal is clearly more dispersive. These linear susceptibilities are taken from several references regarding linear optical properties of Au[1,2] and Si[3–5].

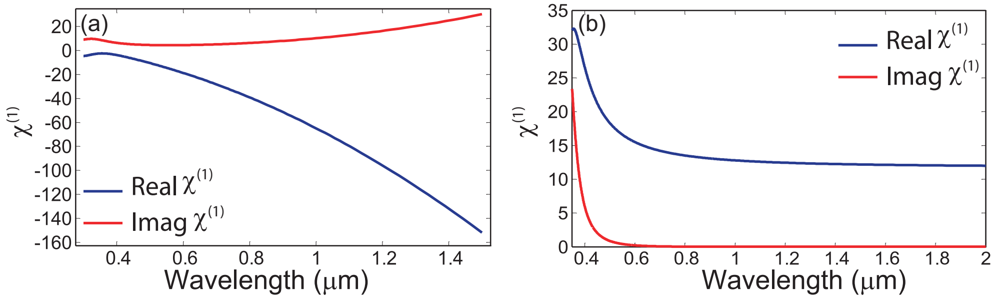

Figure 1. Frequency dispersion of linear susceptibility of Au and Si. (**a**), (**b**) The wavelength dependence of the real and imaginary parts of the linear susceptibility of Au and Si, respectively. The dispersion of the metal is clearly stronger.

Frequency Dispersion of Surface and Bulk Nonlinear Susceptibilities

In order to properly describe the nonlinear optical response of the nanoparticles investigated in our paper, one has to incorporate in the computational analysis the frequency dependence of the surface and bulk nonlinear susceptibilities. This frequency dispersion can be described using the Miller rule[6]. Explicitly, this rule states that the ratio

$$\frac{\chi^{(2)}(\omega_1+\omega_2,\omega_1,\omega_2)}{\chi^{(1)}(\omega_1+\omega_2)\chi^{(1)}(\omega_1)\chi^{(1)}(\omega_2)}=\mathscr{C} \tag{1}$$

is nearly constant. In Eq. (1), $\chi^{(1)}$ is the linear susceptibility, $\chi^{(2)}$ is any of the surface or bulk nonlinear susceptibilities, $\hat{\chi}^{(2)}_{s,\perp\perp\perp}$, $\hat{\chi}^{(2)}_{s,\|\|\perp}=\hat{\chi}^{(2)}_{s,\|\perp\|}$, γ, and ζ, whereas ω_1 and ω_2 are the frequencies of the interacting beams.

As our paper concerns with second-harmonic generation (SHG), $\omega_1=\omega_2\equiv\omega$. The constant $\mathscr{C}$ can be calculated using the corresponding experimental data at an arbitrary reference frequency, ω_r, and thence subsequently used to calculate the full nonlinear dispersions of our materials, Au and Si. In particular, $\mathscr{C}$ is give by

$$\mathscr{C}=\frac{\chi^{(2)}(\Omega_r,\omega_r)}{\chi^{(1)}(\Omega_r)\left[\chi^{(1)}(\omega_r)\right]^2}, \tag{2}$$

where $\Omega_r=2\omega_r$.

The real and imaginary parts of the main bulk nonlinear susceptibility, γ, are presented in Fig. 2. Due to the nature of the Miller rule, the shape of the dispersion curves corresponding to the other nonlinear susceptibilities will remain the same; only the scaling constant $\mathscr{C}$ will change. In our calculations, the reference wavelength was $\lambda_r=810\,\mathrm{nm}$ for Au and $\lambda_r=800\,\mathrm{nm}$ for Si. A comparison of the data plotted in Fig. 2 reveals that for Au the magnitude of the nonlinear susceptibilities increases when the wavelength increases, whereas it decreases in the case of Si.

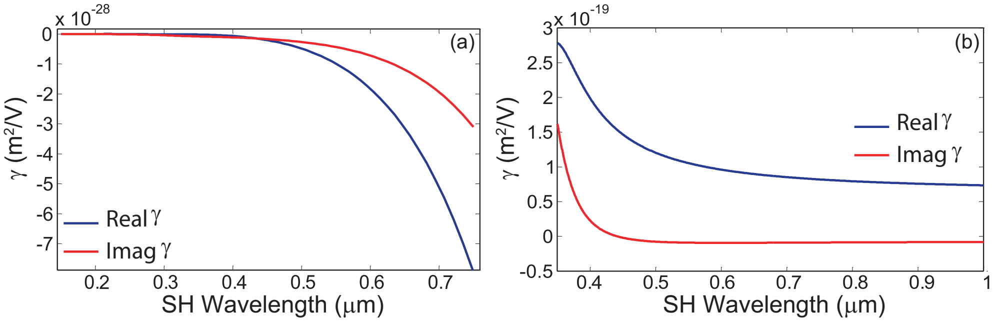

Figure 2. Frequency dispersion of second-order surface and bulk susceptibilities of Au and Si. (**a**), (**b**) The wavelength dependence of the bulk nonlinear susceptibility, γ, of Au and Si, respectively. All nonlinear susceptibilities will follow the same wavelength dependence, but multiplied by a different constant.

Nonlinear Optical Response for Nondispersive Surface and Bulk Nonlinear Susceptibilities

The results presented in the Main Text correspond to the case in which the frequency dispersion of the surface and bulk nonlinear susceptibilities is taken into account. In order to gain a better understanding

of the influence of the dispersion of surface and bulk nonlinear susceptibilities on the conclusions of our study, we have calculated the surface and bulk contributions to the total SHG, as well as the ratio between these two contributions, in the dispersionless case, namely when these nonlinear optical constants are frequency independent. These calculations were performed both for Au and for Si. The values of the surface and bulk nonlinear susceptibilities of Au and Si used in these calculations are given in the Main Text and correspond to the reference wavelengths $\lambda_r = 810\,\text{nm}$ (Au) and $\lambda_r = 800\,\text{nm}$ (Si).

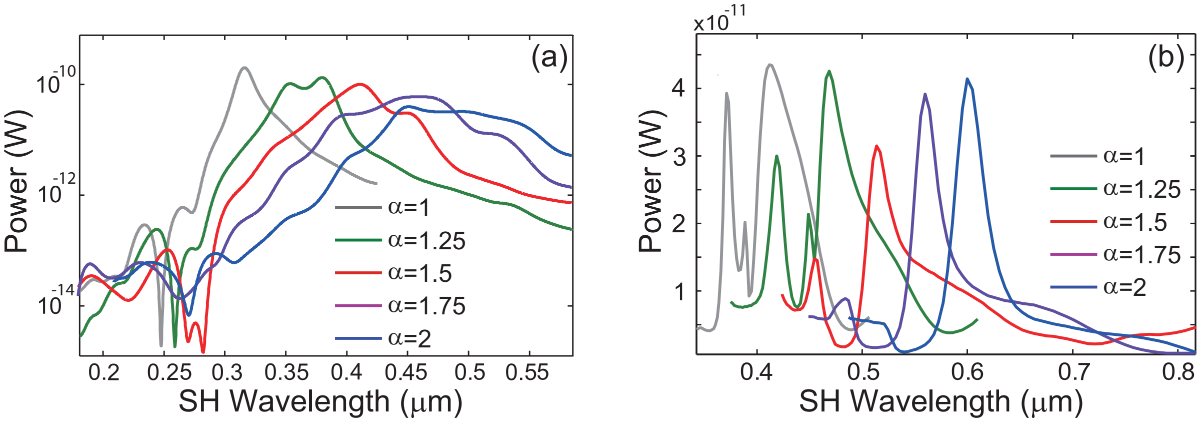

Figure 3. Surface SHG radiated power – dispersionless case. (**a**) The spectra of the power radiated at the second-harmonic by the nonlinear surface sources induced in a cross made of gold, determined for different scaling values, α. (**b**) The same as in (**a**) but calculated for crosses made of silicon. A log scale is chosen for the Au case to help highlight the resonances.

For an easy comparison, the results are plotted in a manner similar to that in the Main Text. More specifically, Fig. 3 is the analog version of Fig. 4 in the Main Text and contains scattering spectra pertaining to the surface contribution to the SHG; Fig. 4 is the analog version of Fig. 5 in the Main Text and contains scattering spectra pertaining to the bulk contribution to the SHG; and finally Fig. 5 is the analog version of Fig. 6 in the Main Text and contains information pertaining to the ratio of the surface and bulk contributions.

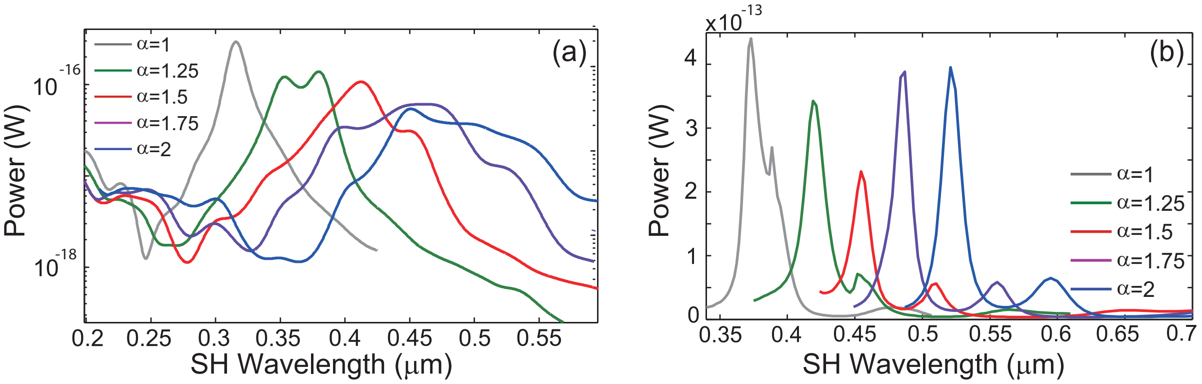

Figure 4. Bulk SHG radiated power – dispersionless case. (**a**) The spectra of the power radiated at the second-harmonic by the nonlinear bulk sources induced in a cross made of gold, determined for different scaling values, α. (**b**) The same as in (**a**) but calculated for crosses made of silicon. A log scale is chosen for the Au case to help highlight the resonances.

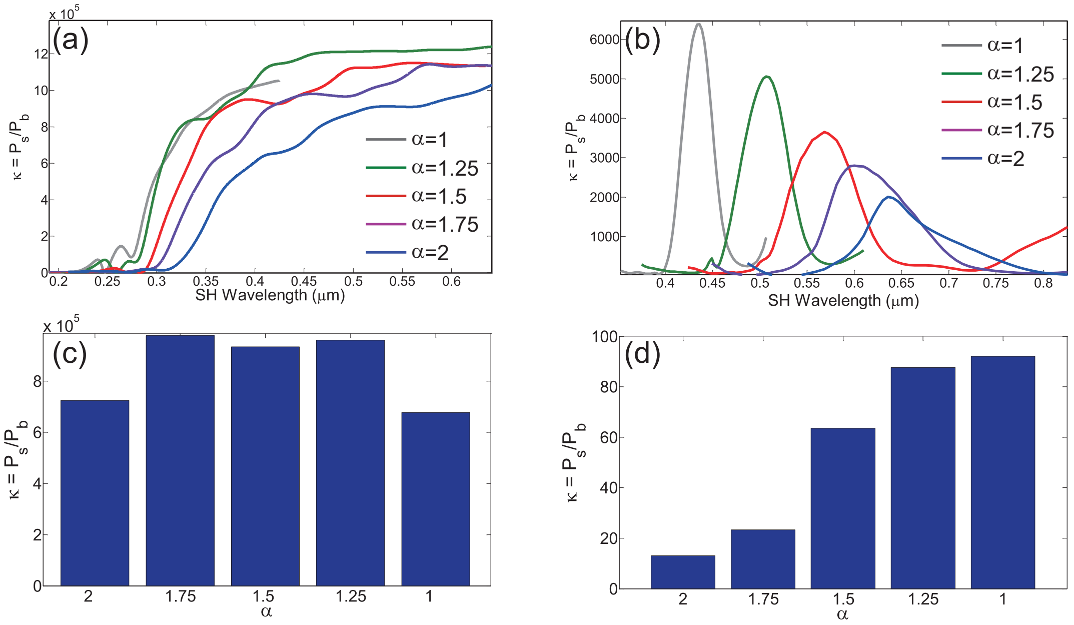

Figure 5. Bulk-to-surface ratio of SHG radiated power – dispersionless case. (**a**) The dependence of the ratio $\kappa = P_s/P_b$ between the SH powers generated by the surface and bulk nonlinear sources induced in a cross made of gold, determined for different scaling values, α. (**b**) The same as in (**a**) but calculated for the crosses made of silicon. (**c**) The value of the ratio κ evaluated at the wavelength at which the bulk contribution is maximum vs. the scaling parameter α, calculated for the crosses made of gold. (**d**) The same as in (**c**) but calculated for the crosses made of silicon.

Differential Scattering Cross-section at the Fundamental Frequency and at the Second Harmonic

An insightful picture of the nature of resonances of nanoparticles is provided by the differential scattering cross-section, as well as the differential cross-sections of the electromagnetic multipoles associated to these resonances. Using a multipolar decomposition of the linear polarization associated to the Au and Si crosses, as per the approach presented in the Main Text, we have calculated the differential scattering cross-sections corresponding to these resonances. The results of this computational analysis, summarized in Fig. 6, clearly demonstrate the specific nature of the main resonances of the two crosses: the Au cross has an electric dipole resonance, whereas the Si cross has an electric dipole resonance, a magnetic dipole resonance, and a mixture between an electric dipole resonance and higher-order resonances.

We also calculated the differential scattering cross-section at the second harmonic, as per Fig. 7. Thus, for resonances corresponding to a wavelength closer to the size of the structure, we expect to see an increased contribution from higher-order multipoles. This is clearly shown in Fig. 7. Whereas the calculated multipoles suggest the dominance of the contribution of the electric dipole in the metallic case, the full differential scattering cross-section shows that the contribution of higher-order multipoles must indeed be considered both for Si and Au scatterers.

Local Field Distribution and Local Field Enhancement

Additional information about the specific nature of various resonances of the nanoparticles investigated in our paper is provided by the near-field distribution. In order to facilitate a better understanding of the characteristics of the near-field distribution corresponding to the main resonances of the silicon cross,

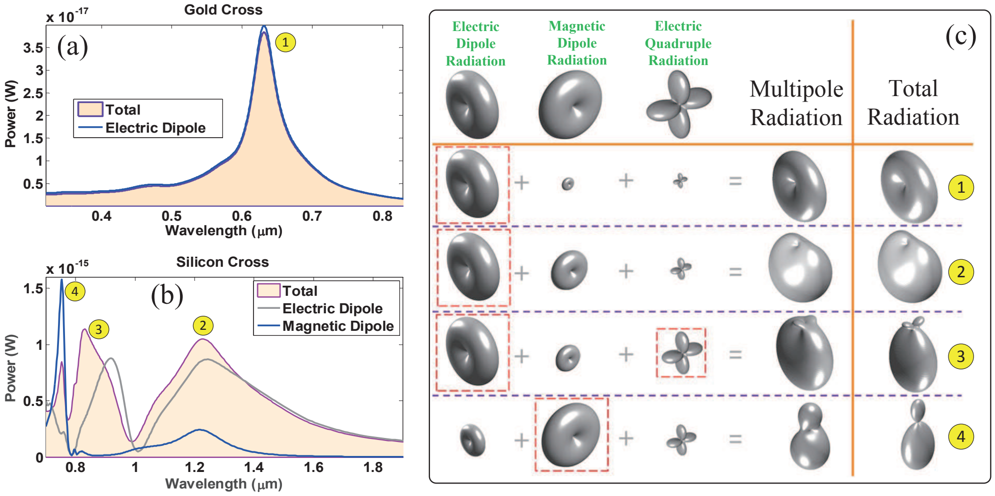

Figure 6. Spectra of radiated power at fundamental frequency and differential scattering cross section. (**a**) Spectra of the total radiated power and the power radiated by the electric dipole of the Au cross with $\alpha = 1$. (**b**) Spectra of the total radiated power and the power radiated by the electric dipole and magnetic dipole of the Si cross with $\alpha = 1$. Resonances 2 and 4 are primarily of electric dipole and magnetic dipole type, respectively, whereas additional terms must be considered to accurately describe resonance 3. (**c**) Differential scattering cross sections corresponding to the main resonances of the Au and Si crosses with $\alpha = 1$, as well as those associated to the electric dipole, magnetic dipole, electric quadrupole, and their sum, calculated at the same resonance wavelengths.

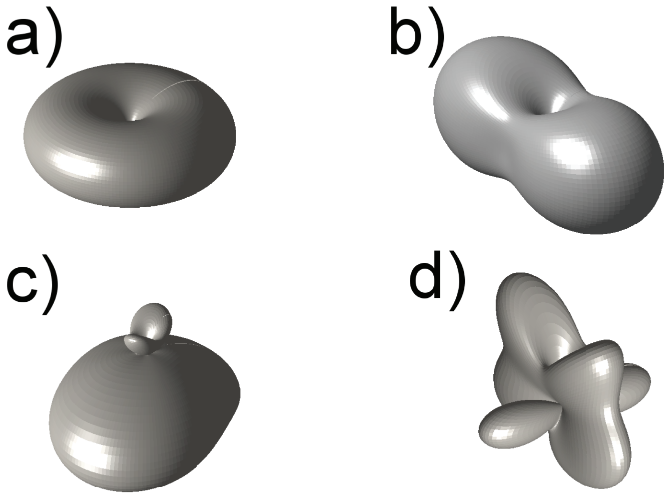

Figure 7. Total differential scattering cross-section at second harmonic. (**a**) Total differential scattering cross-section corresponding to the main resonance at the fundamental frequency ($\lambda = 0.82\,\mu$m) of an Au cross with $\alpha = 1.5$, calculated by summing the contribution of the first three multipoles. (**b**) Full differential scattering cross-section calculated for the same Au cross as in (**a**). The two results are clearly not in agreement, and hence a more rigorous multipole analysis would require more multipoles to be calculated. (**c**) Total differential scattering cross-section corresponding to the electric dipole resonance of a Si cross ($\lambda = 1.44\,\mu$m) with $\alpha = 1.5$. (**d**) Full differential scattering cross-section calculated for the same Si cross as in (**c**). As expected, for the Si cross a larger discrepancy between the results is observed.

denoted in the Main Text of the paper as A, B, and C, we present in Fig. 8 these distributions computed in the main symmetry planes of the cross. As discussed in the Main Text of the paper, resonances A and C correspond to an electric dipole mode and a superposition of a strong magnetic dipole mode and a weaker, higher-order magnetic mode, respectively, whereas the resonance B is the result of a mixture of multipole resonances. At resonance A, an additional contribution from a magnetic dipole mode can be observed, too.

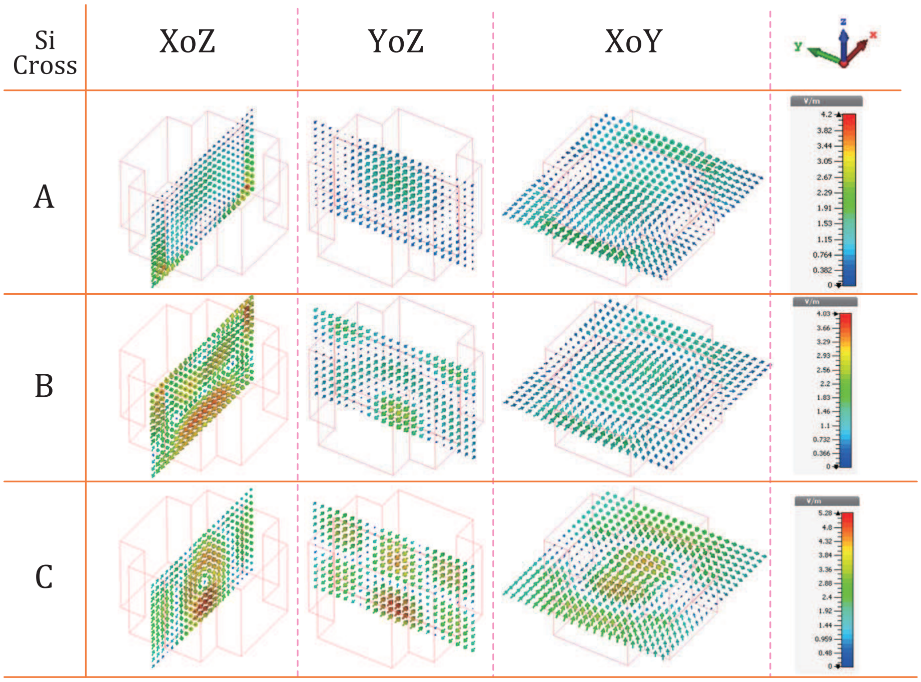

Figure 8. Optical near-field. Near-field distributions computed in the main symmetry planes of the silicon cross, corresponding to the main resonances denoted in the Main Text of the paper as A, B, and C.

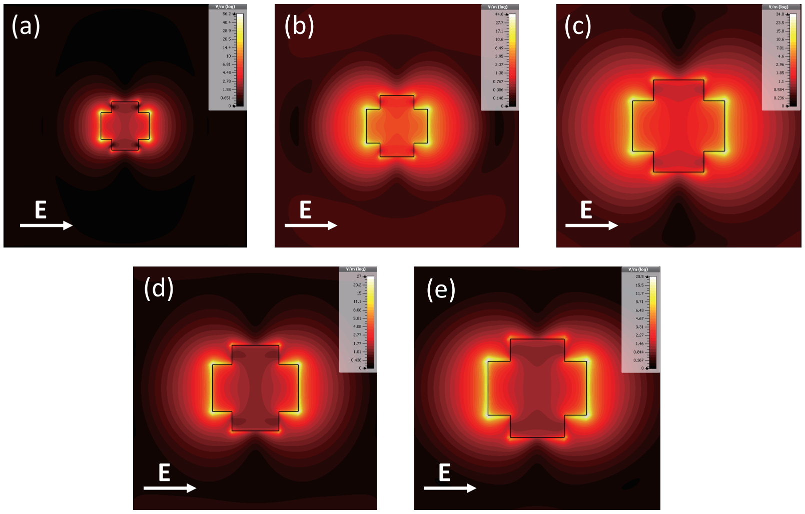

Figure 9. Optical near-field enhancement. (**a**), (**b**), (**c**), (**d**), (**e**) Distribution of the amplitude of the electric field determined for Au crosses with scaling parameter $\alpha = 1, 1.25, 1.5, 1.75, 2$, respectively. The arrow indicates the polarization of the incident wave and the amplitude of the incident field is 1 V/m.

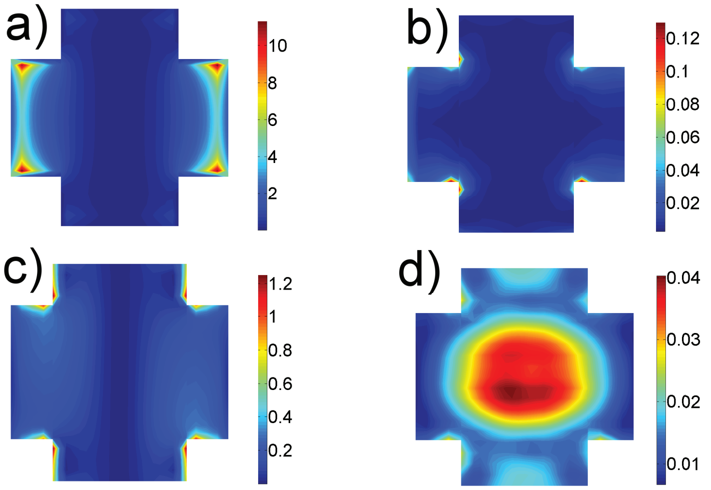

Figure 10. Nonlinear surface and bulk currents. (**a**), (**b**) Amplitude of the surface and bulk nonlinear currents, respectively, corresponding to the main resonance at the fundamental frequency ($\lambda = 0.82\,\mu$m) of the Au cross with $\alpha = 1.5$, multiplied by a factor of 10^5. The surface and bulk nonlinear currents correspond to the top surface and a plane located 2 nm beneath the top surface, respectively. (**c**), (**d**) The same as (**a**), (**b**) but for a cross made of Si and wavelength $\lambda = 1.44\,\mu$m corresponding to the electric dipole resonance at the fundamental frequency. The surface and bulk nonlinear currents correspond to the top surface and the mid-sectional plane (located 110 nm beneath the top surface), respectively.

The SHG is primarily determined by the characteristics of the near-field distribution at the fundamental frequency. In particular, it is expected that the larger the enhancement of the near-field is, the stronger the SHG is. In order to illustrate this idea, we plot in Fig. 9 the spatial distribution of the amplitude of the electric field determined at the resonance wavelength for gold crosses with different size. As discussed in the Main Text, it can be seen that as the size of the cross decreases, the maximum enhancement of the near-field increases, which explains why in the case when the surface and bulk nonlinear susceptibilities are frequency-independent the SHG increases, too [see also Figs. 3(a) and 4(b)].

To further cast light on the SHG characteristics, we plot in Fig 10 the absolute value of the current density, decomposed into the surface and bulk contributions at a wavelength corresponding to a main resonance in the scattering spectrum at the fundamental frequency. As discussed in the Main Text, the surface contribution outweighs that corresponding to the bulk for both materials.

References

1. Werner, W. S. M., Glantschnig, K. & Ambrosch-Draxl, C. Optical constants and inelastic electron-scattering data for 17 elemental metals. *J. Phys. Chem. Ref. Data* **38**, 1013–1092 (2009).
2. Olmon, R. L. *et al.* Optical dielectric function of gold. *Phys. Rev. B* **86**, 235147 (2012).
3. Salzberg, C. D. & Villa, J. J. Infrared refractive indexes of silicon germanium and modified selenium glass. *J. Opt. Soc. Am.* **47**, 244–246 (1957).
4. Edwards, D. F. & Ochoa, E. Infrared refractive index of silicon. *Appl. Opt.* **19**, 4130–4131 (1980).
5. Green, M. A. & Keevers, M. J. Optical properties of intrinsic silicon at 300 K. *Progr. Photovolt.* **3**, 189–192 (1995).
6. Boyd, R. W. *Nonlinear Optics* (Academic Press, 2008).
